# Stability of Dental Implants and Thickness of Cortical Bone: Clinical Research and Future Perspectives. A Systematic Review

**DOI:** 10.3390/ma14237183

**Published:** 2021-11-25

**Authors:** Danilo Alessio Di Stefano, Paolo Arosio, Paolo Capparè, Silvia Barbon, Enrico Felice Gherlone

**Affiliations:** 1 Dental School, Vita-Salute University IRCCS San Raffaele, 20132 Milan, Italy; distefano@centrocivitali.it (D.A.D.S.); gherlone.enrico@hsr.it (E.F.G.); 2 Private Practitioner, 20132 Milan, Italy; 3 Private Practitioner, 20871 Vimercate, Italy; p.arosio@libero.it; 4 Department of Dentistry, Vita-Salute University IRCCS San Raffaele, 20132 Milan, Italy; 5 Section of Human Anatomy, Department of Neurosciences, Padua University, 35121 Padua, Italy; silvia.barbon@yahoo.it

**Keywords:** dental implant, primary stability, secondary stability, osseointegration, cortical bone

## Abstract

Dental surgery implantation has become increasingly important among procedures that aim to rehabilitate edentulous patients to restore esthetics and the mastication ability. The optimal stability of dental implants is correlated primarily to the quality and quantity of bone. This systematic literature review describes clinical research focusing on the correlation between cortical bone thickness and primary/secondary stability of dental fixtures. To predict successful outcome of prosthetic treatment, quantification of bone density at the osteotomy site is, in general, taken into account, with little attention being paid to assessment of the thickness of cortical bone. Nevertheless, local variations in bone structure (including cortical thickness) could explain differences in clinical practice with regard to implantation success, marginal bone resorption or anchorage loss. Current knowledge is preliminarily detailed, while tentatively identifying which inconclusive or unexplored aspects merit further investigation.

## 1. Introduction

Oral rehabilitation through dental implantation has become increasingly important among procedures that aim to replace missing teeth to restore esthetics and the mastication ability of patients [1,2]. Showing a prevalence of success of 90–95% over 10 years of follow-up [3,4,5], endosseous implants must fulfil well-established criteria to be considered completely osseointegrated (i.e., structurally and functionally connected to living bone). These criteria were defined first by Albrektsson and collaborators [6] and then implemented by Misch and colleagues [7]. The latter established that dental implants are successful with marginal bone loss (MBL) <1 mm within the first year and <0.2 mm after the first year from implant placement. Other criteria include the absence of peri-implantitis, implant mobility, discomfort, infection or paresthesia [6]. MBL is, therefore, of paramount importance for the prediction of successful clinical outcomes over the different phases of prosthetic treatment (i.e., implant placement, implant loading, follow-up). MBL is measured by means of intra-oral periapical radiographs obtained by the long cone paralleling method (to minimize distortion) using a customized occlusal bite jig attached to film holders [8]. This method allows measurement of the peri-implant bone level (PBL) at the two sides of the fixture over the course of the treatment, with MBL being defined as the difference between the PBL before implantation and PBL at different intervals postoperatively. Moreover, a scale of implant outcome has been determined to help clinicians to distinguish between success, satisfactory survival, compromised survival and failure of dental implantation [7].

The first prerequisite for the success of dental implantation is represented by achieving sufficient primary stability. This is defined as the absence of mobility of the implant after insertion and is dependent upon mechanical engagement of the fixture with the surrounding bone [9,10,11]. During bone healing, insufficient primary stability can cause excessive micromotion (>50–100 µm) at the bone–implant interface. Such micromotion can interfere with osseointegration and lead to the formation of fibrous scar tissue and hypertrophy of the surrounding trabecular bone [12]. Thus, achieving optimal primary stability prevents the formation of a connective-tissue layer between the fixture and bone. This action ensures secondary stability (also known as “biologic stability”), which is determined by the remodeling and functional regeneration of the bone surrounding the implant (i.e., osseointegration of the implant) [13,14].

Primary stability of the implant has been found to be dependent upon the surgical method (the relationship between the drill size and fixture size) and the microscopic/macroscopic morphology of the implant (i.e., shape, surface roughness) [9,15,16,17,18]. The quantity (thickness) and quality (density) of the bone at the implant site also influences primary stability [17,18,19]. Differences in the outcomes of implant osseointegration may be justified by local differences in the anatomy and morphology of the bone. For example, the lower jaw shows a higher ratio of compact (cortical) bone to cancellous (trabecular) bone in comparison with the upper jaw [18]. Clinical studies have shown longer survival of the implant in the lower jaw than in the upper jaw because, in low-density bone, the primary stability of the implant has been demonstrated to be lower than that in high-density bone [20].

Dental implantation exhibits a high and predictable prevalence of success, but correct assessment of the relation between bone quality, primary stability and osseointegration of implants is still a major challenge. For example, the relationship between a denser thickness of cortical bone and implant stability has been the subject of low-quality clinical reports only, and this has not helped clinicians wishing to use this type of bone to design, prepare or place dental implants. Nevertheless, knowledge of this topic is important to refine the practice of dental implantation, as well as to minimize the risks of its failure.

For this, we aim, through this review, to systematically summarize the current state of the art concerning the relation between cortical bone thickness and implant primary or secondary stability and provide a preliminary assessment of the possible role of cortical bone in achieving and maintaining the stability and osseointegration of dental implants.

## 2. Materials and Methods

### 2.1. Search Strategy

The present systematic review was performed based on the PRISMA statement guidelines [21]. The literature search was carried out by analyzing different electronic databases, such as MEDLINE (PubMed), EMBASE, Cochrane Central Register of Controlled Trials, and Scopus. Eligible articles were searched by using the following keywords and MeSH terms, or combinations of them: “implant stability”, “dental implant stability”, “primary stability”, “secondary stability”, “osseointegration”, “marginal bone loss”, “bone density”, “dental bone density”, “cortical bone”, “cortical thickness”, “cortical bone density”.

The search strategy was adapted to the characteristics of each database to identify studies of interest for this review. The databases were searched for papers and abstracts with no language restriction.

### 2.2. Focus Question

Is there any relationship between cortical bone thickness/density/anchorage and primary implant stability/secondary implant stability/marginal bone loss?

### 2.3. Eligibility Criteria

The following criteria for study inclusion were applied: (1) Participants: individuals undergoing dental implant insertion at any location; (2) Intervention: regular or mini-implant placement, reporting measures of implant stability; (3) Outcomes: the outcome measures were (i) cortical bone thickness/density/anchorage measured on computed tomography/cone beam computed tomography images or assessed by tactile sensations during high-speed drilling, (ii) dental implant stability evaluated by insertion torque (IT) values, resonance frequency analysis (RFA), implant stability quotient (ISQ), Periotest values (PTV) or MBL, (iii) statistical calculation of the correlation between cortical bone thickness/density/anchorage and primary implant stability/secondary implant stability/MBL; (4) Study types: prospective and retrospective clinical studies and cadaver studies.

The exclusion criteria were the following: (1) cross-sectional studies, case series, case reports, pre-clinical studies, in vitro investigations; (2) studies not reporting cortical bone thickness/density/anchorage measures, implant stability measures and statistical correlation analysis; (3) clinical studies not clearly meeting the inclusion criteria.

### 2.4. Study Selection and Data Extraction

Study selection was performed by screening titles and abstracts of articles found through the electronic searches. The full text of all relevant papers was evaluated for inclusion. Articles were selected by considering their compliance with the inclusion criteria.

Data extraction from selected studies was performed by recording the following information: first author, publication year, study design, number and type of dental implants and outcome measurements.

Additional studies of density and quality of bone and measurements for stability of dental implants were also considered to present the state of the art about the topic, as well as to discuss the critical issues addressed by the review.

### 2.5. Risk of Bias Assessment

The risk of bias was estimated for the selected studies according to the Cochrane Handbook for Systematic Reviews of Interventions [22]. The following items were considered and judged: random generation, allocation concealment, blinding of participants and personnel, blinding of outcome assessment, incomplete outcome data and other sources of bias.

## 3. Results

### 3.1. Study Selection

**Figure 1:**
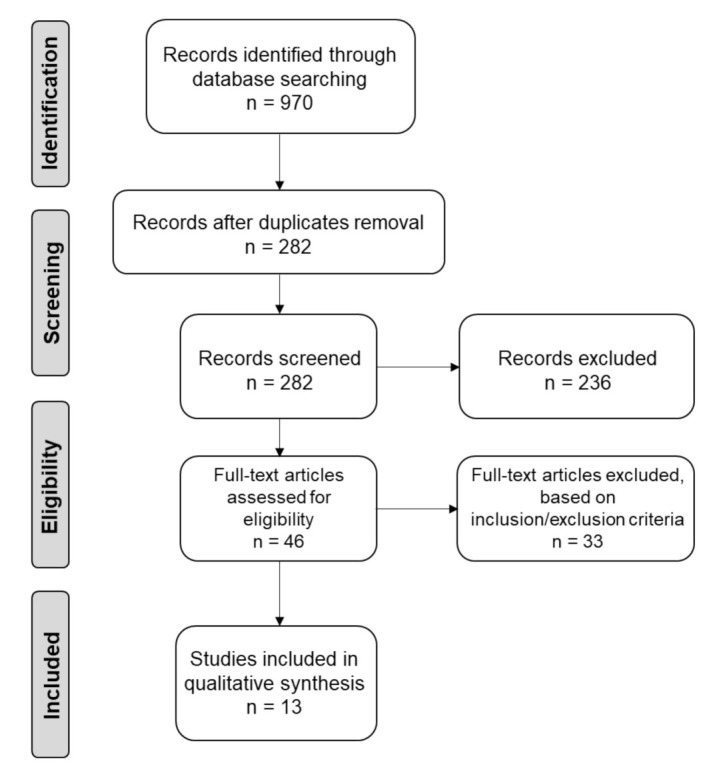
PRISMA flowchart summarizing the selection process of the systematic review.

Database searching first identified a total of 970 records, and 282 of them were maintained after removing duplicates. In the first phase of study selection, screening the title and abstract led to the exclusion of 236 publications. The full texts of the remaining 46 articles were evaluated, and 13 records [23,24,25,26,27,28,29,30,31,32,33,34,35] were found to meet the eligibility criteria and then included in the present study. The flowchart diagram (Figure 1) summarizes the process of study selection.

### 3.2. Study Characteristics

An overview of the characteristics of eligible articles is provided in Table 1. Among the 13 included studies, 8 were prospective clinical trials, 4 were retrospective clinical trials and 1 was a cadaver study. Two studies investigated orthodontic mini-implants, while the remaining 11 trials described the placement of regular implants, with one RCT comparing the insertion of tapered versus cylindrical implants. Cortical bone was evaluated by CT, CBCT and tactile sensations during high-speed drilling. Primary stability was measured by IT, RFA (ISQ) and PTV, whereas secondary stability was measured by RFA (ISQ). MBL was also evaluated after osseointegration of the implants.

### 3.3. Risk of Bias Assessment

**Figure 2:**
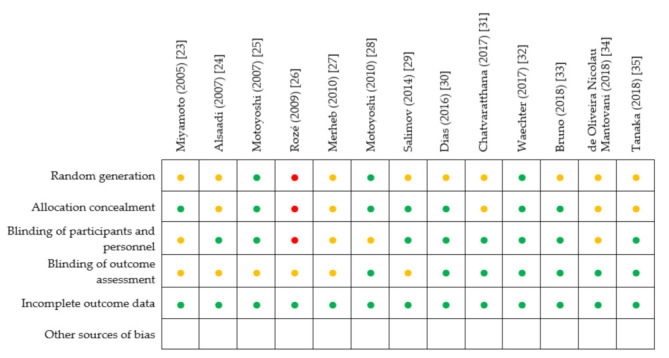
Results of risk of bias assessment for each included study (•: Low risk of bias; •: High risk of bias; •: Some concerns).

A proposed judgement about the risk of bias arising from each selected study was reported about the considered domains described in Section 2.5. Judgement could be *“*Low*”* or *“*High*”* risk of bias, or could express *“*Some concerns*”*. Results of risk of bias assessment are detailed in Figure 2. Overall, the analysis revealed the good quality of the selected studies, with some concerns regarding random generation, allocation concealment and blinding of outcome assessment, which were unclearly reported or missing in some trials.

## 4. Measurements for Stability of Dental Implants

### 4.1. Insertion Torque (IT)

The assessment of primary stability at implantation represents a valid prognostic factor for a successful osseointegration. The primary stability of implants is commonly quantified by a non-invasive clinical method: the insertion torque (IT) test [36]. IT is a parameter that measures the frictional resistance that the fixture encounters while advancing in the apical direction through a rotatory movement on its axis. Peak IT (i.e., the maximal registered IT) is expressed in Newton centimeter (Ncm) units and is predictive of the primary and secondary stability of the implant [37,38,39]. Consensus on the minimum IT needed to achieve osseointegration is lacking. Nevertheless, oral surgeons usually refer to an IT of 20–40 Ncm to establish “ideal” primary stability upon implant placement [40,41]. IT values in this range have been found to prevent adverse micromovements (threshold level between 50 µm and 100 µm) under implant loading, which promotes the osseointegration [42]. Historically, clinical experience has described a linear correlation between primary stability and implant IT up to 50 Ncm. Low IT is associated with protection from biologic and mechanical complications caused by high torsional strengths that may hinder microcirculation due to compression of the surrounding bone [43,44]. This scenario could cause superfluous stress to the bone–fixture system, induce bone necrosis and compromise implant osseointegration [45]. A parallel-group randomized trial by Barone and Colleagues [43] directly compared the clinical outcomes of dental fixtures inserted with regular (<50 Ncm) and high (≥50 Ncm) IT. They demonstrated that a higher IT resulted in peri-implant bone loss and buccal soft-tissue recession, which led to an unsuccessful implantation. They also pointed out that, at implant sites where the cortical bone was more represented (mandible), the use of high IT produced the worst clinical effects. However, recent systematic reviews on this subject underline that studies concerning the association between high IT and marginal bone loss are still inconclusive, and more research is recommended on the matter [46,47,48].

Although IT is considered to be a reliable measure for the primary stability of implants, secondary stability cannot be assessed by a torque ratchet or by using IT-measuring “micromotors”.

### 4.2. Implant Stability Quotient (ISQ)

Another common parameter to measure the stability of an implant is the implant stability quotient (ISQ), which is determined by resonance frequency analysis (RFA) [49,50]. ISQ is measured by a portable, hand-held device (Osstell™, Osstell, Gothenburg, Sweden), which measures magnetic pulses by a transducer (SmartPeg™, Osstell, Gothenburg, Sweden) directly connected to the fixture or prosthetic components. The device places a lateral force upon the implant and calculates the resonance frequency from the electric-response signal. Thus, this method evaluates the “stiffness” and micromotion of the implant–bone complex [38,50,51]. The resonance frequency obtained by Osstell™ (Osstell, Gothenburg, Sweden) is translated into ISQ in an automatic manner, which ranges from 1 to 100. Higher ISQ values indicate greater implant stability. An ISQ of 57–82 denotes successfully osseointegrated implants; an ISQ <50 predicts a high risk of implant failure [52,53].

The IT measurement can be obtained only upon implant placement. ISQ can be recorded in all phases of prosthetic treatment: upon fixture insertion, during the healing phase and even after the prosthesis has been loaded [54]. Hence, ISQ allows for implant stability to be assessed over time and represents a reliable measure of primary stability and secondary stability.

### 4.3. Periotest™

Although the most widespread methods for the evaluation of implant stability are IT measurement and RFA, another non-invasive device called the Periotest™ (Denti, Budapest, Hungary) can be used. Originally designed to determine tooth movement in a quantitative way, the Periotest value (PTV) assesses the increased stiffness of the implant–bone continuum over time [55]. The range of PTV recorded for a clinically stable fixture is dependent upon the characteristics of the tissues around it (bone in the case of osseointegration and fibrous tissues in failed implants). Even minimal clinical mobility is considered a symptom of implant failure, so PTV evaluation may be of clinical interest [56]. However, PTV results are related strongly to the direction and position of excitation, so this evaluation method does not always measure a precise biomechanical parameter. Hence, Osstell™ (Osstell, Gothenburg, Sweden) is usually preferred for the assessment of implant stability [51,56].

### 4.4. Dynamic Parameters of Primary Stability

IT and ISQ measure the bone–implant interaction in a different way, therefore, they provide different information [57,58]. To clarify this ambiguity, some clinicians have suggested to evaluate primary stability by measuring the insertion energy (IE), that is the amount of energy required to insert the implant into the site of interest [59,60,61]. Preliminary results have demonstrated that IE may be more reliable than RFA or IT to achieve acceptable primary stability even in softer bone [59] and is more reproducible at quantifying primary stability enhancement provided by under-preparation [62]. However, the relationship between IE, RFA and IT has still to be investigated in depth [61].

A “surgical micromotor” has been developed recently: it allows to record the implant insertion-related parameters, such as instantaneous, average and peak IT values, as well as the insertion torque/depth integral [63]. The latter (under the conditions that the insertion rotational speed is constant and the implant threads are equally spaced, two conditions encountered quite often in clinical practice) is proportionally related to IE. Insertion torque/depth integral has been shown to correlate significantly with the bone-to-implant contact (BIC) in a study on bovine ribs and in a clinical setting [64,65]. Insertion torque/depth integral has been demonstrated to provide an operator-independent and reliable assessment of PS and to show greater sensitivity to bone-density variations than primary stability IT and ISQ [63]. Insertion torque/depth integral has also been shown to provide: (i) reliable information about BIC and primary stability even in tapped/undersized sites [66]; (ii) different information than IT about the immediate primary stability of implants of different shapes [39].

## 5. Density and Quality of Bone

The quantity and quality of the host bone are determined by the crestal cortical bone thickness and the inner cancellous bone density, as well as by their relative distribution in the implant recipient site. Poor bone quantity and density are the main risk factors for fixture failure because they are related to excessive resorption of bone and impaired healing processes [13]. Remarkably, the bone density at the implantation site has been shown to proportionally affect IT and ISQ: a higher density of local bone corresponds to a higher value of IT and ISQ [67,68]. This finding implies that clinical assessment of bone quality upon implantation plays a part in determining primary stability and subsequent osseointegration. Thus, it appears relevant to develop measurements of bone quality as a determinant for the successful outcome of endosseous implantation.

Bone quality was classified first by Lekholm and Zarb [69] based on the morphology and distribution of cortical bone and trabecular bone. Four classes of residual alveolar bone were distinguished: type 1 (large homogenous cortical bone); type 2 (dense medullar bone surrounded by a thick cortical layer); type 3 (dense medullar bone surrounded by a thin cortical layer); type 4 (sparse medullar bone surrounded by a thin cortical layer). Lekholm and Zarb reported that the best outcome in implant therapy is obtained with a suitable amount of cortical thickness surrounding a cancellous region (type 1 and type 2 bone). Subsequently, a classification system developed by Misch [70] was based on the perception of bone quality during drilling, which also provided comparative materials of different resistance to drilling to aid classification. This system identified five density groups (D1–D5) associated with specific locations of the jaw and tactile analogs (Table 2).

However, intraoperative drilling resistance cannot be considered an objective quantitative assessment of bone quality. Computed tomography (CT) offers the opportunity to rely on a preoperatively quantitative determination of bone density that is not dependent upon the expertise of the operator [71]. CT axial images have 260,000 pixels, and each pixel corresponds to a CT number (Hounsfield unit: HU), which is associated with the density of the tissues within the pixel. Generally, higher CT numbers are associated with denser tissues. Hence, the Misch classification of bone density can be correlated to a range of HU by CT evaluation (Table 3) [70].

Thus, the use of CT became an objective method for the preoperative quantification of bone density, with several studies corroborating the relationship between CT measurements and the primary stability of the implant [29,72,73,74]. However, concerns about radiation exposure to patients make CT a nonviable option for the routine measurement of bone density.

To avoid exposure to high levels of radiation during CT, cone beam computed tomography (CBCT), which is designed specifically for the head and neck, has eclipsed CT [75]. CBCT is a three-dimensional imaging technique with high resolution that allows the collection of volumetric data on jaw bones and teeth with relatively low radiation doses and costs [75,76]. CBCT is employed routinely to plan implant placement if it might damage delicate anatomic structures (e.g., inferior alveolar nerve or maxillary sinuses). However, the reproducibility of bone-density measurements using CBCT is limited because the calibration of CBCT machines is usually brand-dependent and not known to the user [77,78,79]. Most surgeons, therefore, still assess bone density according to the D1–D5 Misch classification [70,80], as modified by Trisi and Rao [81] by subjective intraoperative perception at drilling.

Several studies have positively correlated a higher prevalence of failure to implant placement into D4 bone. Conversely, good osseointegration is associated with implants placed into D1–D3 bone, thereby suggesting that D3 is the “ideal” type of bone for the adequate primary stability of implants [20,82]. Overall, bone quality is regarded to be a key factor in planning implantation and the surgical procedure, as well as for defining the healing period and implant loading [83].

### Intraoperative Measurement of Bone Density

The same IT-measuring micromotor described in Section 4.4 also enables quantitative intraoperative and site-specific bone-density evaluation during implant-site preparation. This micromotor can measure bone density during implant-site preparation by means of a specific probe. Such density measurement is based on the assumption that the resistance encountered during threading is a good index of site-specific bone quality [36,84], as shown by Friberg et al. in studies on pig ribs and jaw autopsy specimens [85,86]. Bench tests on blocks of polyurethane foam simulating cancellous bone have shown that the average IT measurement provided by the micromotor correlates with the actual block density in a significant way, and enables measurement of the average statistical error introduced by the device-operator system during bone-density assessment [87]. The same tests enabled the creation of calibration curves for the device with and without irrigation. The average IT measurements have also been shown to correlate significantly with histomorphometric bone-density measurements of bovine ribs [88]. If used in humans, the IT-measuring micromotor provides operator-independent bone-density measurements and correctly discriminates between the anterior and posterior areas of both arches [89,90]; it has also been used recently to draw a position-by-position topologic map of the bone density of cancellous bone for both the upper and lower jaw [91].

## 6. Thickness of Cortical Bone and Implant Stability

### 6.1. Thickness of Cortical Bone in the Upper and Lower Jaw

“Bone quality” is defined as the thickness of cortical bone and density of cancellous bone, as well as the cortical/cancellous ratio. Bone quality is a crucial factor for implant osseointegration. Nevertheless, most studies have focused on measuring cancellous bone density, whereas the influence of cortical bone thickness on the achievement of an implant’s primary and secondary stability has been poorly investigated. Primary implant stability is influenced even by the ratio between cortical and cancellous bone, i.e., the greater such a ratio the greater the ISQ as well as the insertion torque. In particular, some authors speculate that the latter may be more affected by the cortical/cancellous ratio than the ISQ [13,26,27,31,92,93,94,95].

Cortical bone thickness is considered to be relevant for achieving implant stability. This is true both for regular implants, which interact with the crestal cortex, and for orthodontic mini-implants, whose stability may be influenced by the buccolingual cortical bone width. Indeed, the coronal and buccolingual cortical thickness are interrelated [31]; thus, studying how cortical bone thickness affects the stability of regular implants may be informative concerning its effect on that of mini-implants and vice versa.

The first systematic studies dealing with the determination of cortical bone thickness for dental implantation date back to the early 2000s, when Schwartz-Dabney and Dechow [96] compared the bone properties of mandibles of edentulous cadavers with those of dentate patients. They pointed out that tooth loss was associated not only with ridge resorption but also with significant microstructural changes in mandibular cortical bone in terms of thickness, elasticity, shear moduli, the anisotropy and orientation of the axis of maximum stiffness. Subsequently, a cadaver study by Katranji and colleagues [97] contributed to the definition of the average thickness of cortical bone in different regions of the upper and lower jaw. Confirming a previous investigation [96], Katranji and colleagues concluded that the average cortical thickness was higher in dentate (1.6–2.2 mm) than in edentulous (1.0–2.1 mm) maxillae and mandibles, with the thinnest area in the anterior upper jaw and the thickest area in the posterior lower jaw.

In 2006, Deguchi and co-workers [98] used CT in orthodontic patients to evaluate the mean thickness of the cortical plate in the buccal and lingual regions (mesial and distal to the first molar and distal to the second molar) and in the premaxillary region at two levels. Based on those measurements, the authors determined that, during orthodontic treatment, the best placement for miniscrew insertion might be mesial or distal to the first molar, where cortical bone is thicker and the distance from the intercortical bone surface to the root surface is higher. The optimal size of the miniscrew should be <1.5 mm in diameter and 6–8 mm in length. In parallel, evaluations of patients who received mini-implants anchored in posterior buccal alveolar bone demonstrated that the average thickness of cortical bone ranged from 1.09 mm to 2.12 mm in the upper jaw and 1.59 mm to 3.03 mm in the lower jaw. Because lower jaw cortical bone is significantly thicker than that of the upper jaw, the lower jaw suffices as a site for mini-implant anchorage, whereas the upper jaw might not be sufficient. Furthermore, cortical bone was shown to be thinner in women than in men in the region of the upper jaw mesial to the first molar [99].

**Figure 3:**
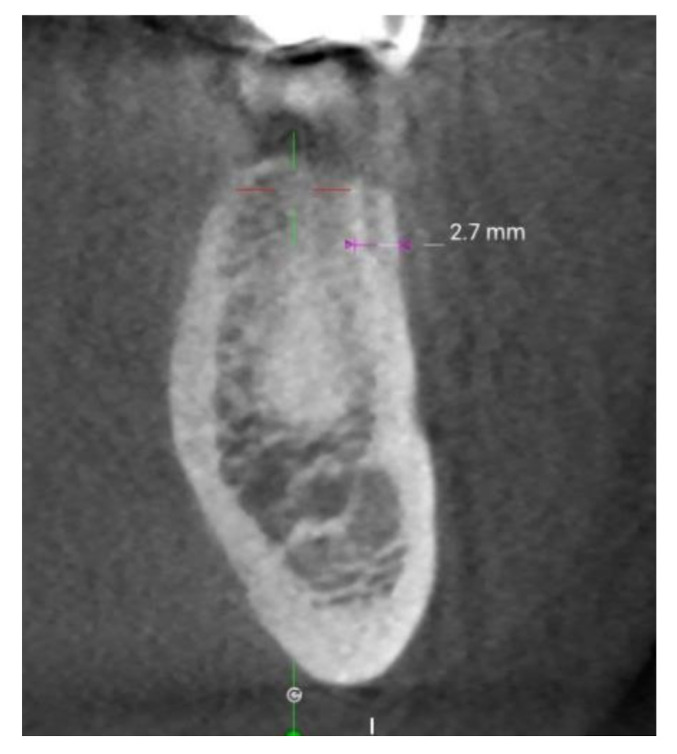
Cortical bone thickness can be easily measured on CBCT scans. The picture shows an axial section of a lower jaw. By using a dedicated software, the surgeon draws a measuring line across the cortical bone at the site to be measured; the software—in its measurement mode—provides the thickness reading directly on the screen.

More recently, with the emergence of dental CBCT methods, patient sample sizes have been increased to augment the statistical robustness of measurements of cortical bone thickness undertaken directly at the preoperative site. Indeed, cortical thickness can be measured directly and quite easily on CBCT scans by using widely available software tools, as shown in Figure 3.

The first clinical study using CBCT images to measure the crestal cortical bone thickness at dental-implantation sites in different jawbone areas was reported by Ko and collaborators [100]. Considering 661 dental-implantation sites, statistical analyses of CBCT measurements revealed that crestal cortical bone thickness (mean ± SD) varied considerably between surgery sites in the four areas of the jawbone, decreasing in the order: posterior lower jaw (1.07 ± 0.47 mm) > anterior lower jaw (0.99 ± 0.36 mm) > anterior upper jaw (0.82 ± 0.30 mm) > posterior upper jaw (0.75 ± 0.35 mm). These results of the distribution of cortical bone thickness in the two arches were confirmed by a similar study by Gupta et al. [101] based on preoperative CBCT measurements at 780 implant sites. They reported the highest thickness in the posterior lower jaw (1.18 mm), followed by the anterior lower jaw (1.08 mm), anterior upper jaw (0.82 mm) and posterior upper jaw (0.76 mm), with significant differences among regions. The difference between sexes was not significant [101]. Overall, such data may provide clinicians with a reference for conducting dental-implant surgery.

With the analogous purpose to guide optimal orthodontic mini-implant placement, characterization of cortical bone was conducted by CT rather than CBCT, and considered 60 high-resolution scans of the maxilla from patients unrelated to dental-implant treatment [102]. That quantification study showed that the density and thickness of cortical bone increased significantly from the coronal (2 mm) to the apical (8 mm) areas of alveolar bone. The average thickness and density of cortical bone were found to be significantly higher in the palatal side rather than the buccal side, with the anterior maxillary region showing the greatest difference. The thickness and density of bone was positively correlated with BMI and age. Bone density (but not bone thickness) was shown to be associated with sex, data that were in accordance with the work from Gupta and colleagues [101] but not with results from Ono and collaborators [99].

Hence, a preoperative evaluation of cortical bone thickness at the implant site appears to be favorable to patients in terms of longer survival, but clinical research measuring this parameter is needed.

### 6.2. Cortical Bone and Primary Stability of Implants

#### 6.2.1. Regular Implants

Given the importance of local bone quality for dental-implant outcome, the first clinical study based on the hypothesis that a thicker cortical layer would improve regular implants’ primary stability was reported by Miyamoto and collaborators [23]. They evaluated 50 edentulous patients who were subjected to a preoperative CT for quantitative imaging of cortical bone thickness. Before radiography, diagnostic radiographic templates were made by incorporating radiopaque indicators. A total of 225 implant insertions were realized, and RFA was undertaken to measure implant stability upon placement by recording ISQ. Statistical analyses of the collected data demonstrated a significant linear correlation (r = 0.84, *p* < 0.0001) between cortical bone thickness and ISQ.

A retrospective study on 298 patients also detected a significant relationship between cortical bone thickness and the primary stability of fixtures [24]. Cortical bone and trabecular bone were assessed subjectively by tactile sensations during high-speed drilling, as experienced by the clinician during surgery and referring to the classification by Lekholm and Zarb [69]. Based on that premise, an evaluation scale was established, ranging from grade 1 (very thick cortical bone) to grade 3/4 (thin or very thin cortical layer). In parallel, bone quality was evaluated objectively during implant insertion by means of an electronic device to measure torque force. Finally, the stability of the implant–bone continuum was assessed by RFA and by Periotest. ISQ and PTV were found to correlate significantly with grades of cortical bone (*p* = 0.02 and *p* < 0.0001, respectively) [24].

Underlining the importance of CT before implant surgery, a clinical trial conducted by Merheb and co-workers [27] determined the correlation between CT parameters and implant stability. To this end, different measures of bone quality (i.e., HU values and coronal cortical thickness at osteotomy sites) were collected preoperatively using CT in 24 patients who presented a fully edentulous maxilla and who received a total of 136 dental fixtures. In parallel, the primary stability of implants was evaluated by RFA upon the placement and loading of implants. A linear relationship was found between cortical bone thickness measured by CT and RFA (*p* < 0.05) upon insertion and loading [27].

Similar preoperative CBCT and subjective evaluations of bone quality during drilling were undertaken in the prospective randomized trial by Waechter et al. [32]. They searched for a relationship between the primary stability parameters (IT and ISQ) of 20 tapered and 20 cylindrical implants placed in the posterior mandible. Linear measurements of receiver sites were undertaken with specific preoperative software tools on multislice/CBCT images to measure the height of cortical bone. For both types of implants, osteotomy was performed through a conventional sequence of drilling without considering under-preparation of the implant site. Besides demonstrating no significant differences between tapered and cylindrical geometries for any outcome variable, the results suggested that bone-site characteristics can influence IT and implant stability. The initial stability (as measured by IT) was directly related to cortical bone height, whereas ISQ seems to be dependent on the availability of cancellous bone [32].

Chatvaratthana and co-workers [31] compared ISQ values obtained from RFA with parameters obtained from CBCT images from 16 patients who received 19 implants into posterior maxillary and mandibular regions. They observed a strong correlation between ISQ and the crestal cortical bone thickness surrounding the implant site (*p* < 0.001).

The ISQ correlated with the cortical bone thickness even when this was measured on the buccal or lingual side 3 mm below the ridge (*p* = 0.018). When the vestibular/lingual cortical bone thickness was measured more in depth (6 or 9 mm below the ridge), no correlation was observed (*p* > 0.05). The authors explain this finding by the fact that the further the cortical bone from the implant the lesser it contributes to stabilize it.

Moreover, the same authors demonstrated that the ratio of the cortical to cancellous bone thickness at 3 mm was significantly related to the ISQ, confirming the reliability of this parameter as an index of bone quantity and quality surrounding the implant site.

Similarly, to provide clinicians with specific instructions about the best surgical method and type of fixture, Bruno and colleagues [33] (75 patients, 269 implants) confirmed the correlation between primary stability of the implant measured by RFA and HU values detected for coronal–buccal (r = 0.302; *p* = 0.020) and middle–lingual (r = 0.295; *p* = 0.023) maxillary sites. Simultaneously, IT showed a positive correlation with cortical bone thickness at the middle of the ridge (ρ = 0.196; *p* = 0.032). In accordance with previous research, a retrospective study by Tanaka and colleagues [35] found a correlation between implant stability and cortical bone thickness. That work involved 113 patients and a total of 229 fixtures placed in both arches, with bone augmentation undertaken in some cases. The thickness of cortical bone at the site of implant insertion was evaluated preoperatively by CT (except for cases of bone grafts and immediate implant placement). For each implant site, RFA was performed in three directions, and the lowest value was recorded. Observing significantly higher mean ISQ results in the lower jaw group than in the upper jaw group, as well as in the non-augmentation than in the augmentation group, a weak positive correlation was observed between cortical bone thickness and primary stability of the fixture (*p* < 0.01) [35].

The predictive value of CT in orthodontic treatment was demonstrated by Salimov and colleagues [29], who analyzed a total of 65 fixtures placed in 17 patients. First, the bone density at recipient sites was recorded preoperatively through CBCT. Then, bone quality was assessed subjectively by the surgeon, according to resistance to drilling and referring to the index created by Lekholm and Zarb [69]. In particular, during drilling, the surgeon scored the bone quality by tactile sensation concentrating on two criteria. First, the surgeon focused on cortical-layer thickness, discriminating the stiffness variation while passing through cortical bone to trabecular bone. Cortical bone thickness ≥1 mm was considered “thick”, whereas <1 mm was considered “thin”. Second, once trabecular bone had been entered, the surgeon evaluated its compactness, defining it as “dense” or “fine”. Finally, during surgery, the peak IT was recorded using a digital torque meter and RFA was undertaken using Osstell™ immediately after implant insertion. Collected data demonstrated a strong correlation (r = 0.791, *p* < 0.001) between HU values derived by CBCT and bone types distinguished according to the index created by Lekholm and Zarb [69] on the basis of cortical bone thickness and trabecular bone patterns. A significant correlation was observed also between bone-density measurements using CBCT and IT/ISQ, showing that evaluation of the bone quality (also in terms of cortical bone thickness) allowed prediction of the optimal PS.

Finally, the key role of cortical bone thickness in the primary stability of implants was demonstrated by a cadaver study evaluating 22 implants inserted through a strict clinical protocol into the maxillae and mandibles of three partially edentulous people. Bone structure was investigated by CT, whereas primary stability of the implant was measured by RFA using Osstell™. After calculation of the bone histomorphometric parameters, they were correlated with recorded ISQ values, which ranged from 50% to 70% depending on the specimens and sites. A sole correlation was found between ISQ and cortical bone thickness, whereas a relationship was not found between the histomorphology of trabecular bone and ISQ. According to those results, a thick cortical bone was associated with high implant stability and, thus, high loading capacity [26].

Interestingly, the influence of the anchorage of cortical bone upon implant stability was considered by retrospective evaluation of primary stability values (IT during implant surgery and ISQ immediately after) and CBCT of 33 patients and a total of 165 surgical procedures. The final sample included 97 fixtures, which were divided into three classes: with apical cortical bone contact; with bicortical bone contact (apical and cervical regions); with cervical cortical bone contact. Higher IT during implant placement and higher ISQ (*p* < 0.05) were recorded for fixtures with bicortical anchorage, whereas monocortical implants (apical and cervical) showed similar results (*p* > 0.05). IT and ISQ were found to be affected by cortical bone contact, but a significant correlation was not observed between IT and ISQ. Hence, higher IT did not necessarily lead to higher ISQ [34].

#### 6.2.2. Orthodontic Mini-Implants

The association between cortical bone thickness and the primary stability of mini-implants was also investigated in two clinical studies by Motoyoshy and colleagues [25,28]. The first study comprised four males and 28 females who received 11 and 76 mini-implants (1.6 mm in width and 8 mm in length), respectively, in the posterior alveolar bone after CT of the insertion site. A successful outcome of mini-implants implied that orthodontic force could be applied for ≥6 months without pain or clinically detectable mobility. The prevalence of implant success was: (i) 87.4% and cortical bone thickness was significantly higher in the successful group (*p* = 0.015); (ii) significantly higher in the group with IT of 8–10 Ncm. Although a relationship between implant stability upon placement and the width/height of the peri-implant bone was not established, Motoyoshy and colleagues determined that the prepared site should have a cortical bone thickness ≥1.0 mm and IT should be limited to 10 Ncm to improve the chance of implant success [25]. In the second study, the stability of 134 mini-implants was evaluated by recording peak IT upon placement and removal, after having measured cortical bone thickness at the preparation sites by CT. The average IT upon placement and removal was ~8 Ncm and ~4 Ncm, respectively. The two parameters were not correlated significantly, whereas IT upon placement (but not upon removal) was found to be related significantly to cortical bone thickness in the upper jaw [28].

The clinical studies mentioned above established an important correlation between primary stability of the implant and cortical bone thickness at the insertion site.

### 6.3. Cortical Bone and Secondary Stability of the Implant/MBL

The relation between cortical bone thickness and secondary stability of the implant has not been studied deeply. A retrospective study by Tanaka and colleagues [35] investigated secondary stability in 113 patients (229 total implants) by RFA and ISQ, whereas the thickness of cortical bone at the insertion site was assessed preoperatively by CT. Mean ISQ after osseointegration was 75.99 ± 6.23, with implants showing significantly higher mean ISQ if placed into mandibular bone rather than maxillary bone, thereby suggesting a weak positive relation between cortical bone thickness and secondary stability of the fixture.

Conversely, a correlation between cortical bone thickness and ISQ, or MBL changes, were described by Dias and co-workers [30]. Evaluating a final sample of 31 patients (57 implants), ISQ and MBL determined by standardized periapical radiographs were registered at different phases of orthodontic treatment: implant insertion, uncovering/loading stage, and at 1-year follow-up. Those results are not in accordance with studies reporting significant relation between cortical bone thickness and implant stability [23,27]. Different techniques of measuring CT images and a more in-depth assessment of the implant–cortical bone interaction, in relation also to the cortical bone preparation, might explain these controversial results, as well as the checkered evidence concerning high IT at insertion and late MBL.

### 6.4. Cortical Bone and Implant Sizing

Cortical bone thickness could be an interesting variable to be considered when undersizing of the implant site needs to be decided. In fact, over-compression of the cortical layer during an undersized drilling protocol could produce trauma to cells and tissues, thereby leading to MBL and compromising implant osseointegration [103]. During undersized implant placement, high compression of the bone is associated with biomechanical events named microcracks, which are expected to cause plastic bone deformation, ischemic necrosis and bone resorption [103]. Indeed, studies on sheep models reporting the histological investigation on peri-implant tissues after undersized drilling, demonstrated bone resorption activity within the cortical layer [104].

Clinical studies reporting on the relation between cortical bone, fixture sizing and implant stability are currently lacking, even though this is considered in daily clinical practice. Furthermore, investigations on synthetic or animal bone models have been highly controversial. In vitro experiments on artificial bone have reported that undersized drilling protocols into thicker cortical bone can lead to higher IT upon implant placement [19,105]. In particular, under-preparation of the implant site has been found to affect IT more significantly than standard drilling in specimens with a cortical layer of thickness 0–1 mm. However, the effect of the undersized surgical approach on IT is less evident if the thickness of the cortical layer is ≥2 mm [19]. Conversely, pre-clinical studies have revealed no influence on IT between undersized and standard protocols if implants are placed in dog radius diaphysis [106], proximal tibia [107] or lower jaw [108], corresponding to highly corticalized bone.

Additionally, clinical evidence needs to be collected about cortical bone resorption, which could be caused by under-preparation of the implant site and the consequent effects on dental-fixture stability. Although it has been suggested that bone could tolerate high levels of compression [103], above a certain threshold negative biologic responses are expected, such as massive bone necrosis and resorption, which may impair bone remodeling and the healing process, as well as the final osseointegration of the implant.

### 6.5. Cortical Bone and Peri-Implantitis

Peri-implantitis is a major cause of dental-fixture failure. It is a common complication due to the inflammation of hard and soft tissues surrounding the fixture, which may lead to hemorrhage, bone loss and fixation failure [109]. Besides the placement of implants into sites with a history of failed endodontic or apicoectomy procedures, the most likely cause of peri-implantitis is surgical-site preparation. In fact, excessive heating of the bone during osteotomy, as well as bone microfracturing for incorrect drilling protocols or overloading of the implant may result in development of the inflammatory conditions associated with bacterial infiltration [110,111,112].

In this context, the cortical layer at the implant site may have an important role: it is sensitive to thermal necrosis and mechanical microfractures where bacterial microorganisms infiltrate, causing inflammation, bone resorption and, finally, implant failure. Therefore, strategies for the prevention of peri-implantitis should take advantage of the assessment of cortical bone thickness at the surgical site. Previous works on implants placed into healed failed endodontic areas have shown that peri-implantitis probably occurs if apical cortical bone is thin or if apical osseous fenestration is present. In these conditions, bacterial colonization may break through thin or non-existent bone, reaching the facial soft tissue and creating a lesion at the surgical site [113]. However, clinical research directly correlating cortical bone thickness and the prevalence of peri-implantitis is lacking.

## 7. Conclusions and Future Perspectives

This systematic review has several limitations, including the scant literature found about the correlation between cortical bone and implant stability, as well as the methodological variability of the included studies. However, it may allow for the drawing of some preliminary conclusions.

It is well known that a rigorous assessment of bone quality—i.e., the thickness of the cortical layer and its spatial and quantitative relation with cancellous bone—is fundamental before implant treatment because it is a key factor when predicting implant stability. However, the literature assessed in this preliminary review seems to indicate that bone density alone is taken into account more often than cortical bone thickness for predicting implant treatment, while the specific thickness of cortical bone at the site of surgery might provide valuable insights into the primary stability of the implant. This possibility should be the subject of more accurate, systematic reviews, followed by appropriate metanalyses, as well as of clinical studies evaluating the relationship between cortical bone thickness, cortical bone preparation and implant stability. Indeed, these studies appear to be lacking, even if the presence and thickness of a cortical bone layer were indeed shown to correlate to increased primary implant stability. Moreover, very little consideration seems to have been given to the influence of cortical bone on secondary stability of the implant and MBL. Should these relations be found to exist and be robust, it would be desirable to setup preoperative procedures that take cortical bone thickness into account, and the evaluation of cortical bone thickness by three-dimensional cone beam radiography might become routine diagnostic practice during the planning of implant treatment. Indeed, a deeper knowledge of the average values for cortical-layer thickness (as well as the inter-patient variability of this characteristic) could aid the site selection and preparation of implants.

Furthermore, very few and controversial studies have been found on the effect of different cortical preparations, even if these may help in achieving adequate stability, in relation to the short- and long-term MBL as well as the corresponding implant survival and success rate. Thus, filling this knowledge gap in the near future is of paramount importance. Moreover, studies focused on the effect of cortical bone thickness in medically compromised patients shall be carried out to gain more knowledge concerning these sub-groups of people. Finally, retrospective clinical research on possible correlations between cortical bone thickness, cortical bone preparation and the incidence of peri-implantitis might help to improve the prognosis and long-term functionality of dental fixtures.

We preliminarily conclude that not only bone density but also cortical bone thickness might become an important predictive parameter during preoperative implant assessment. The presence of a cortical layer, as well as its thickness, should in fact be knowingly considered as key factors in programming implant stability. To this end, appropriate, systematic, in-depth literature analyses and clinical researches should be implemented.

## Figures and Tables

**Table 1:** Characteristics of the included studies (*n* = 13).

First Author (Publication Year)	Study Design	Intervention	Outcomes		
			Evaluation of Cortical Bone	Evaluation of Dental ImplantStability	Statistical Correlation
Miyamoto (2005) [23]	Prospective clinical study	225 dental implants (diameter, 3.5 mm; length, 8-9-11-13-15 or 17 mm); (maxilla 98, mandible 127)	Preoperative CT scans	Primary stability measured by RFA (ISQ)	Yes (r = 0.84, *p* < 0.0001)
Alsaadi (2007) [24]	Retrospective clinical study	761 Mark III TiUnite™ implants(maxilla 386, mandible 334)	Tactile sensations during high-speed drilling	Primary stability measured by IT, ISQ and PTV	Yes Significant relationship between ISQ, PTV and cortical bone grades (*p* = 0.02 and *p* < 0.0001, respectively)
Motoyoshi (2007) [25]	Prospective clinical study	87 mini-implants (1.6 mm wide and 8 mm long) placed in the posterior alveolar bone	Preoperative CT scans	Primary stability measured by IT	No Cortical bone thickness of at least 1.0 mm and IT up to 10 Ncm improve implant success rate
Rozé (2009) [26]	Cadaver study	22 implants into maxillary and mandibular sites	CT	Primary stability measured by RFA (ISQ)	Yes (*p* = 0.003)
Merheb (2010) [27]	Prospective clinical study	136 dental implants into the upper jaw (diameter, 3.3 or 4.1 mm; length, 6, 8, 10, 12 or 14 mm)	Preoperative CT scans	Primary stability measured by RFA and PTV	Yes (*p* < 0.05)
Motoyoshi (2010) [28]	Prospective clinical study	134 mini-implants placed into posterior maxillary and mandibular sites (diameter, 1.6 mm; length, 8 mm)	CT	Primary stability measured by IT upon implant placement and removal	YesSignificant correlation between cortical bone thickness and placement torque in the upper jaw (r = 0.392, *p* < 0.05)
Salimov (2014) [29]	Prospective clinical study	65 dental implants (diameter, 3.4, 3.8 or 4.3 mm; length, 12 mm) (maxilla 44, mandible 21)	CBCT Tactile sensations during high-speed drilling	Primary stability measured by IT, RFA (ISQ)	YesSignificant correlation between IT, ISQ and cortical bone density (r = 0.935, *p* < 0.001 and r = 0.888, *p* < 0.001, respectively)
Dias (2016) [30]	Prospective clinical study	57 dental implants (maxilla 22, mandible 35)	CT images	Implant stability measured by RFA (ISQ) MBL measured by periapical radiographs at the 1-year follow-up	No No significant relationship between MBL changes and cortical thickness (r = −0.029; *p* = 0.832) and between cortical thickness and ISQ (r = 0.145; *p* = 0.292)
Chatvaratthana (2017) [31]	Prospective clinical study	19 implants (diameter, 5 mm; length, 9 mm) inserted into posterior maxillary and mandibular sites	CBCT	Primary stability measured by RFA (ISQ)	Yes (*p* < 0.001)
Waechter (2017) [32]	Prospective RCT	20 tapered implants (diameter, 4.6 mm; length, 10 mm) and 20 cylindrical implants (diameter, 4 mm; length, 10 mm) into the posterior mandible	Tactile sensations during high-speed drilling	Primary stability measured by IT and ISQ	Yes IT was directly related to cortical bone height (r = 0.32; *p* = 0.0441). ISQ seems to be dependent on cancellous bone availability (r = 0.32; *p* = 0.0471).
Bruno (2018) [33]	Retrospective clinical study	269 implants (mean diameter, 4.36 ± 0.64 mm; mean length, 13.08 ± 1.71 mm) (maxilla 149, mandible 120)	CT	Primary stability measured by IT and ISQ	YesPositive correlation between IT and cortical bone thickness at the middle of the ridge (ρ = 0.196; *p* = 0.032)
de Oliveira Nicolau Mantovani (2018) [34]	Retrospective clinical study	97 implants into mandibular sites, divided into 3 classes: (1) with apical cortical bone contact; (2) with bicortical bone contact; (3) with cervical cortical bone contact	CBCT	Primary stability measured by IT and ISQ	Yes IT values and ISQ are influenced by cortical bone anchorage (i.e., bicortical bone anchorage led to higher IT and ISQ, *p* < 0.05)
Tanaka (2018) [35]	Retrospective clinical study	229 dental implants (diameter range, 3.0–5.0 mm; length range, 6–13 mm) (maxilla, 111; mandible, 118)	CT	Primary and secondary implant stability measured by RFA (ISQ)	Yes Weak positive correlation between cortical bone thickness and primary/secondary implant stability (*p* < 0.01)

Abbreviations: CBCT, cone beam computed tomography; CT, computed tomography; ISQ, implant stability quotient; IT, insertion torque; MBL, marginal bone level; PTV, Periotest values; RCT, randomized clinical trial; RFA, resonance frequency analysis.

**Table 2:** Classification of bone density by Misch according to clinical drilling resistance of bone.

Bone Density	Description	Tactile Analog	Location
D1	Dense cortical bone	Oak wood	Anterior lower jaw
D2	Porous cortical bone and dense trabecular bone	Spruce wood	Anterior lower jaw Posterior lower jaw Anterior upper jaw
D3	Thin and porous cortical bone and thin trabecular bone	Balsa wood	Posterior lower jaw Anterior upper jaw Posterior upper jaw
D4	Thin trabecular bone	Styrofoam™	Posterior upper jaw
D5	Non-mineralized bone (unsuitable for implantation)	-	-

**Table 3:** Classification of bone density by Misch correlated to a range of Hounsfield units by CT evaluation.

Bone Density	CT Evaluation	Description
D1	>1250 HU	Dense cortical bone of the anterior lower jaw
D2	850–1250 HU	Porous cortical and coarse trabecular bone in the anterior/posterior mandible and anterior upper jaw
D3	350–850 HU	Thin cortical and fine trabecular bone of the posterior lower jaw and anterior/posterior upper jaw
D4	150–350 HU	Fine trabecular bone of the posterior upper jaw
D5	<150 HU	Immature non-mineralized bone

## Data Availability

Data sharing not applicable.
